# Aluminum

**Published:** 1904-04-15

**Authors:** E. Bumgardner


### ALUMINUM.

BY E. BUMGARDNER, D.D.S.

The first time aluminum was placed on exhibition at a world's fair a small ingot of it was labeled "The Silver from Clay," and the popular idea grew up that it is similar to silver. In color, in its fusing point, in being a good conductor of heat and electricity and in ductility and malleability, aluminum does resemble silver, but the parallel cannot be carried far. Contact of a sulphur compound immediately gives silver a black surface, while aluminum is not tarnished in this manner. Silver is a noble metal, which means that it can be obtained from its ores by simply heating them, while aluminum defied the efforts of chemists to reduce it fifty years after it had been named as the metallic base of alumina. In hardness aluminum is more like zinc. Its specific gravity is much lower than any of the most familiar metals, being only 2.56, while silver is 10.53, gold 19.3 and platinum 21.5. One qualitiy of aluminum is suggested by the thought of the first article ever made of it—a baby-rattle, which was made for the baby Prince of France.

Aluminum is never found in the metallic state in Nature. It is, however, the most abundant of all the metals. It is estimated that its compounds make up one-twelfth of the earth. Every clay bank is an aluminum mine, as clay is the silicate of the metal. The oxide, $Al_2O_3$, is found in various forms. The crystalline form is the mineral corundum, of which emery, the ruby, sapphire, oriental amethyst and oriental topaz are varieties. The silicates, fluorides, phosphates, carbonates and other compounds are represented by hundreds of minerals, many of which are unattractive, while many others are gems worthy of the lapidary art. The topaz of Brazil, which when properly cut so closely resembles the diamond, is a pure silicate of aluminum. The turquoise is a phosphate of aluminum. Garnets exist in a score or more of varieties and colors, all compounds of aluminum. Cryolite, from which the metal has until lately been chiefly obtained, is a double fluoride of aluminum and sodium.

Like most metals, aluminum can be made softer and more pliable and malleable by annealing, but as it melts before it becomes red hot it cannot be annealed as gold or platinum. The safest method is that recommended by Richards, of coating with stearine or tallow the piece to be annealed, and removing from the flame as soon as this is burned off the metal.

Aluminum is not affected by any of the single acids except hydrochloric, which is its true solvent. Unlike most metals, it is readily attacked by alkaline solutions, such as caustic potash. In consequence of its low specific gravity, freedom from tarnish, non-poisonous qualities and ease of working, aluminum is a valuable metal for many purposes, and its range of usefulness will gradually be extended. Its use in dentistry, however, will always be limited on account of its tendency to disintegrate by the action of the alkaline fluids of the mouth. This fault is not so noticeable now that the metal can be obtained more nearly pure than formerly, but it can probably never be entirely eliminated. Another undesirable quality in aluminum as a base for artificial dentures is its want of elasticity.

The one obstacle that, more than any other, stands in the way of the use of aluminum, especially for our purposes, is the difficulty of soldering it. Our experience in working gold is of little value to us here. In soldering gold we can judge with considerable accuracy by the color of the red-hot metal when we are in danger of melting the piece to be soldered, which is not the case when the fusing point of the metal which we are working is below that which produces redness. The low specific gravity of aluminum is another disadvantage in soldering. Gravitation does not seem to overcome the tendency which all molten substances have to assume the globular form. Any perfect solder is made chiefly from the metal to be united. Solder composed largely of aluminum is not heavy enough to flow down into the joint as gold solder does. Then aluminum is deficient in a quality which I do not know to how describe except by comparing with capillary attraction. It does not "wet" easily, and is said to be an unsuitable material for racing boats for this reason. So the melted solder does not spread out and run along the joint as we want it to do, and in order to cause the solder to come into contact with the metal as it must do before adhesion can take place, it must be pressed or tamped into position. The composition of the solder and the flux to be used with it are two other hard questions. The solders most commonly used have been composed of from 80 to 92 percent of zinc. By adding a little tin the proportion of zinc may be diminished, but zinc remains an important constituent. A very good solder for aluminum and one which will not undergo rapid change in the mouth may be made as follows:

R. Aluminum ..........60 parts.
Zinc.................10 parts.
Phosphor-tin..........30 parts.

Organic compounds all the way from vaseline to lemon juice have been recommended for use as fluxes. One of the best that I know of and one that works very well with the solder just mentioned is stearic acid or common stearine. —*Western Dental Journal.*

